# Neuroprotective effects of *Lippia javanica (Burm.F.) Spreng.* Herbal tea infusion on Lead-induced oxidative brain damage in Wistar rats

**DOI:** 10.1186/s12906-021-03471-3

**Published:** 2022-01-04

**Authors:** Zubair Suleman, Godwill A. Engwa, Mathulo Shauli, Hannibal T. Musarurwa, Ndinashe A. Katuruza, Constance R. Sewani-Rusike

**Affiliations:** 1 Department of Human Biology, Faculty of Health Sciences, Walter Sisulu University PBX1, Mthatha, 5117 South Africa; 2 Department of Biological and Environmental Sciences, Faculty of Natural Sciences, Walter Sisulu University PBX1, Mthatha, 5117 South Africa

**Keywords:** Lead, Oxidative stress, Inflammation, Apoptosis, Neuronal damage, Polyphenols

## Abstract

**Background:**

Though *Lippia javanica (Burm.f.) Spreng* antioxidant activity has been demonstrated, its effect in protecting the brain from lead (Pb)-induced oxidative damage is unknown. This study investigated the effect of *L. javanica* against Pb-induced oxidative stress, inflammation, apoptosis and acetylcholinesterase activity in rat’s brain.

**Methods:**

*L. javanica* herbal tea infusion was prepared, its phytochemical constituent was revealed by liquid chromatography-Mass spectrometer (LC-MS) and was administered simultaneously with Pb. Four groups of male Wistar rats (*n* = 5/group) were used: control received distilled water; Pb-acetate group received 50 mg Pb/ Kg bodyweight (bw), treatment group received 50 mg Pb/ Kg Pb-acetate + 5 ml/kg bw *L. javanica* and *L. javanica* group received 5 ml/Kg bw of *L. javanica* tea infusion only. After 6 weeks of treatment, oxidative status, acetylcholinesterase activity, inflammation and apoptosis was assessed in brain tissue which was also histologically examined.

**Results:**

Mean brain and heart weight was reduced (*p* < 0.05) while liver and spleen weights were increased (*p* < 0.05) in Pb exposed animals but were prevented by *L. juvanica* treatment. Treatment with *L. javanica* increased *(p < 0.05)* overall brain antioxidant status (glutathione and superoxide dismutase activities) and reduced lipid peroxidation (*p* < 0.05) compared to the Pb exposed animals. Pro-inflammatory cytokine tumour necrotic factor-alpha, pro-apoptosis Bax protein and anticholinesterase activity were reduced (*p* < 0.05) in Pb-*L. javanica* treated animals compared to the Pb exposed group. Histological examination confirmed neuroprotective effects of *L. javanica* as evidenced by reduced apoptosis/necrosis and inflammation-induced vacuolization and oedema in the hippocampus. The *L. javanica* treatment alone had no detrimental effects to the rats. LC-MS analysis revealed *L. javanica* to be rich in phenolics.

**Conclusions:**

This study demonstrated that *L. javanica,* rich in phenolics was effective in reducing Pb-induced brain oxidative stress, inflammation, apoptosis, acetylcholinesterase activity and neuronal damage.

## Introduction

Lead (Pb) is a toxic heavy metal widely distributed across environments all around the world with no beneficial roles in biological systems [1]. Exposure to Pb has been a problem of global concern for decades especially in children [2]. Pb poisoning is known to alter the body function as it can affect the hematologic, hepatic, cardiovascular, reproductive, gastrointestinal and neurological systems in the body [3]. As such, Pb has been reported to affect speech, nerve conduction and hearing, as well as cause weight loss, hyperactivity, intestinal discomfort, vomiting, constipation, and muscle aches. Pb poisoning can equally lead to anemia, paralysis, nephropathy, convulsions or death [4]. In pregnancy, Pb can affect the foetus causing intrauterine deaths, miscarriages and/or low birth weight [5].

Neurological damage induced by Pb toxicity is reported to be a base for multiple disorders such as Parkinson’s and Alzheimer’s diseases [6]. Pb has the ability to pass through the blood-brain barrier due to its calcium ion-substituting ability to damage the prefrontal cortex, cerebellum and hippocampus [7]. The disruption of the brain barrier by Pb causes albumin to enter the tissues of the central nervous system (CNS), resulting in increased intracranial pressure, edema, and encephalopathy [8]. Pb exposure in the brain affects neurotransmitters including cholinergic, dopaminergic and glutaminergic systems [9]. One of the characteristic features of Pb intoxication in the brain is impairment of the action of acetylcholinesterase (AChE) in cholinergic nerve cells transmission [10]. The central cholinergic system is essential for the regulation of cognitive functions. As such, cholinergic receptor agonists and inhibitors of AChE are used to control endogenous acetylcholine levels to overcome cognitive deficits [11].

Pb neurotoxicity also promotes pro-inflammatory effects in the brain. Pb is known to induce increase gene expression and secretion of interleukin 6 (IL-6), tumour necrotic factor-α (TNF-α) and transforming growth factor β1 (TGF-β1) leading to increased inflammation in the brain [11]. Inflammatory processes have been observed in the pathogenesis of Parkinson’s, Alzheimer’s diseases and in multiple sclerosis [12]. Also, Pb toxicity is known to cause apoptotic neurodegeneration [13]. Studies have shown Pb to increase mRNA and protein levels of apoptotic factors including caspase and Bcl-2 Associated X Protein (Bax), evidence that Pb increases neurodegeneration [14].

Pb toxicity induces oxidative stress by generating reactive oxygen species (ROS) such as superoxide (O^2−^), hydroperoxides (HO_2_^•^) and hydrogen peroxide (H_2_O_2_), as well as by depleting the antioxidant defence systems [15]. Glutathione (GSH) is an important antioxidant that directly or indirectly scavenges ROS. Also, antioxidant enzymes including superoxide dismutase (SOD) and catalase (CAT) are important ROS scavengers. However, these enzymes can be rendered inactive or reduced by Pb [15]. Failure of the body to meet the homeostatic requirement due to overwhelming ROS level results in the occurrence of oxidative stress characterised by oxidative damage of proteins, nucleic acid and membrane lipids (lipid peroxidation) [16]. Cellular by-products of oxidative damage also stimulate inflammation and cell death.

In recent years, studies conducted on the use of antioxidants as an intervention for Pb-induced neurotoxicity and oxidative stress has shown success. Gallic acid, a phenolic compound of natural origin attenuated locomotor damage and brain oxidative stress induced by Pb exposure [17]. Also, geinstein was shown to alleviate Pb-induced neurotoxicity through the involvement of multiple signalling pathways in vitro and in vivo [18]. Plants rich in phenolic compounds are known to play an important role as antioxidants which enhances their medicinal properties. One of such plant is *Lippia javanica (Burm.f.) Spreng.* which has been shown to possess antioxidant activities in in-vitro studies [19–21]. Traditionally, parts of *L. javanica* have been used medicinally for the treatment of respiratory ailments such as colds, coughs, asthma and tuberculosis [22]. It has also been reported in the treatment of gastrointestinal tract disorders such as abdominal pain [23] which is a common symptom of Pb poisoning. Although *L. javanica* has been shown to possess higher antioxidant capacity as well as better free radical scavenging activity than a known antioxidant, rooibos (*Aspalathus linearis*) in vitro [24], it remains unknown whether the in vitro antioxidant properties of *L. javanica* can translate to in vivo neuroprotective effects of Pb-induced toxicity against oxidative stress, inflammation, apoptosis and acetylcholinesterase activity. Therefore, the aim of this study was to investigate the effectiveness of *L. javanica* in attenuating Pb-induced toxicity in rat’s brain.

## Materials and methods

### Preparation of tea infusion

The *Lippia javanica (Burm.f.) Spreng.* herbal tea was purchased from Harare, Zimbabwe. One hundred millilitres (100 ml) of boiled deionised water was added to 5 g of dry *L. javanica* leaves followed by stirring with a magnetic stirrer for 10 min then steeping for 30 min and strained with a fine mesh tea strainer followed by vacuum filtration through Whatman No. 1 filter paper [25]. The tea infusion was prepared weekly and used for animal treatments.

### Experimental animals and ethics statement

All methods were performed in accordance with the study protocol of relevant institutional, national, and international guidelines, regulations and legislation required for plants and animal studies. This study was conducted in accordance with the ethical guidelines of Animal Care and Use and the Animal Research: Reporting of In Vivo Experiments (ARRIVE) guidelines. Ethical clearance with approval number: [060/2016] was obtained from the Faculty of Health Sciences, Walter Sisulu University ethical committee on the 14th of November 2016 before commencement of study. Twenty (20) Wistar, adult male rats (270 g–340 g) which were 10 weeks old were procured from South African Vaccine Producers (Johannesburg, South Africa) and housed in a temperature-controlled (20 °C-24 °C) animal house in plastic cages with open, steel tops, with each cage housing 5 rats. The rats had free access to rat pellet feed (Epol, SA) and tap water which was topped up twice daily to ensure constant availability. The cages were cleaned and new bedding was added twice a week. The general health status of the rats was observed daily before and after treatment and their body weights was measured and recorded once every 2 weeks. The rats were handled in a humane manner in keeping with the guidelines as specified by the National Council of the Society for the Prevention of Cruelty to Animals (SPCA) and the South African National Standard: The care and use of animals for scientific purposes (2008) [26]**.** The rats were euthanized humanely by CO_2_ inhalation.

### Animal treatments

Male Wistar rats were divided into 4 treatment groups of 5 rats per group. All treatments were administered by oral gavage using a rat feeding needle (18G) attached to a 5 ml syringe. Treatments were given for 5 consecutive days per week for a total of 6 weeks. The experimental groups were:

1. Control group: 1 ml of distilled water
2. Pb-acetate only: 50 mg of lead acetate per kg bodyweight (bw) in 1 ml volume.
3. Pb-acetate + *L. javanica*: 50 mg/kg bw of lead acetate and 5 ml/Kg bw *L. javanica* tea infusion each in 1 ml volume.
4. *L. javanica* only: 5 ml/kg bw of *L. javanica* tea infusion in 1 ml volume

Pb-acetate dosage was selected based on a previous study [27] which is equivalent to 32 mg/kg Pb, below the rat oral LD_50_ of 56 mg/kg [28], while the dosage for *L. javanica* is 1 cup human dose equivalent as reported previously for rooibos tea [29].

### Terminal procedures

At the end of the 6-week treatment period, all the rats were terminated by CO_2_ inhalation. Vital organs (heart, liver, kidneys and spleen) were harvested, cleaned of excess adhering tissue, weighed and percentage of body weight calculated (Organ weight index) for comparison across treatment groups. The brains were removed by partial decapitation, weighed and then halved medially. Half of the brain was immediately frozen at − 70 °C (Skadi Green Line Ultra-Low freezer, UK) for homogenization and biochemical analyses. The other half brain tissue was fixed in 10% buffered formalin (Associated Chemical Enterprises, Johannesburg, South Africa) for histology.

### Tissue homogenization

Brain tissue (0.2–0.4 g) was thawed and homogenized in 0.01 M phosphate buffered saline, pH 7.4 (PBS; Sigma Aldrich, USA) to attain a concentration of 1:10 (w/v) using Potter-Elvehjem glass tissue homogenizer. The resultant homogenates were placed in glass test tubes on ice and centrifuged for 10 min at 3000 rpm at 4 °C (Eppendorf 5810 R, Germany) and the resultant supernatants were collected in Eppendorf microtubes for biochemical analysis.

### Biochemical assays

#### Total antioxidant capacity

Trolox equivalent antioxidant capacity (TEAC) assay was used to measure the total antioxidant capacity (TAC) in the brain tissue samples reflective of radical scavenging activity of antioxidants according to the method of Arnao and colleagues [30]. In this assay, the antioxidant capacity of a sample was determined based on the antioxidant reaction that reduces the blue ABTS^•+^ radical to its neutral, colourless form, ABTS (2, 2′-azino-bis-(3-ethylbenzthiazoline-6-sulphonic acid). Trolox (6-hydroxy-2, 5, 7, 8-tetramethylchroman-2-carboxylic acid) was used for construction of the standard curve. Absorbance was read at 734 nm in a spectrophotometer (Phoenix-2000 V UV-VIS, Biotech Engineering Management Co. Ltd. (UK)). The assay was run on 50 μl of brain tissue homogenate in duplicate. TAC of the sample was calculated using the linear equation from the standard curve and were expressed as mg/ml Trolox equivalent (TE).

#### Determination of reduced glutathione concentration

Reduced glutathione (GSH) was determined as previously reported by Owens and Belcher [31]. The assay utilised Ellman’s reagent (5, 5 dithiobis 2-nitrobenzoic acid) which reacts with reduced GSH resulting in a yellow colour chromophore, 5- thionitrobenzoic acid (TNB) with intensity proportionate to the GSH concentration in samples and standards, read at 415 nm. The GSH concentration of samples was calculated using the linear equation from the standard curve and expressed in μMol GSH/g tissue.

#### Superoxide dismutase activity

Superoxide dismutase (SOD) activity in brain tissue samples was measured using a commercial assay kit (Cayman, Ann Arbor, USA) as per manufacturer’s instructions. SOD activity of the samples was calculated using the linear equation from the standard curve and expressed in U/mL.

#### Lipid peroxidation

The lipid peroxidation of the homogenised brain tissue samples was determined by the TBARS (thiobarbituric acid reactive substances) assay according to the method of Mallick and colleagues [32]. The principle of this assay relies on the reaction of 2-thiobarbituric acid with malondialdehyde (MDA), a product and marker of lipid peroxidation, at 100 °C to yield a pink chromophore whose absorbance was read at 532 nm in a spectrophotometer (Phoenix-2000 V UV-VIS, Biotech Engineering Management Co. Ltd. (UK)). The MDA concentration was calculated using molar extinction coefficient of MDA and expressed in μM.

#### Determination of acetylcholinesterase activity

The acetylcholinesterase (AChE) activity was determined using the colorimetric assay as previously described by Ellman and colleagues [33]. Briefly, brain homogenate was mixed with Ellman’s reagent (5,5′-dithio-2-nitrobenzoic acid) followed by the addition of the enzyme substrate, acetylthiocholine. The yellow colour change in absorbance/min during a 5 min interval was measured at 405 nm in a microplate reader (BioRad, CA, USA). The change in absorbance/min was expressed as enzyme activity in μmoles of acetylthiocholine hydrolyzed/ min/ gram of wet brain tissue (μmoles/min/g tissue).

#### Quantification of tumour necrosis factor-alpha (TNF-α) and Bax protein

The pro-inflammatory cytokine TNF-α (pg/ml) and pro-apoptosis protein Bax (ng/ml) were measured using commercial enzyme-linked immunosorbent assay (ELISA) kits (Elabscience® USA catalogue E-EL-R0019 and E-EL-R0098 respectively) according to manufacturers’ protocol.

### Histology

Brain tissue fixed in 10% buffered formalin was subjected to standard procedures for fixing, washing, dehydration and paraffin embedding as previously described by us [34]. Following fixing of the brain tissue, 5-μm sections were cut and mounted on clean slides. Slides were stained with haematoxylin and counterstained with eosin for light microscopic examination. Images were taken using a digital Leica DMD108 microscope (Wetzlar, Germany).

### Liquid chromatography-mass spectrometer analysis of *Lippia javanica*

Leaves of *L. javanica* were dried at room temperature and ground with a pestle in a mortar into powder. The dried leaf powder (2 g) was soaked overnight with 15 mL of 50% methanol in deionised water containing 1% formic acid. It was then extracted in an ultrasonic bath (0.5 Hz, Integral Systems, RSA) for 60 min at room temperature and centrifuged (Hermle Z160m,) at 3000 g for 5 min. The supernatant was transferred into vials for LC-MS analysis. The extract was analysed in an ultra-performance liquid chromatography (UPLC) (Waters, Milford, MA, USA) connected to a Waters Synapt G2 Quadrupole time-of-flight (QTOF) mass spectrometer (MS) as previously reported by us [35]. Polyalanine was used for calibration and calculations according to Rautenbach et al. [36]. Samples were run in triplicate and results presented as relative abundance (%).

### Statistical analysis

Data was analysed using GraphPad Prism Version 5 statistical package and presented as mean ± standard error of the mean (SEM) in tables and figures. One-way analysis of variance (ANOVA) was used to compare means between groups followed by Tukey’s multiple-comparison test. A *p* ≤ 0.05 was considered statistically significant.

## Results

### Effect of *Lippia javanica* on organ weights

All organ weights were expressed as percent of body weight. Pb treatment resulted in increased liver and spleen weight indices (*p* < 0.05) and reduced heart and brain weight indices (*p* < 0.05) compared to the controls. These Pb associated detrimental effects on liver, heart, spleen and brain were prevented by treatment with *L. javanica* except the kidney which was increased compared to controls. Treatment with *L. javanica* alone had no effect on organ weights (Table 1).

**Table 1:** Mean organ weight expressed as organ indices (%) relative to body weights after 6 weeks of treatment

Organs	Organ Weight Indices (% bw)			
	Con	Pb	Pb + Lip	Lip
Heart	0.4 ± 0.04	0.35 ± 0.01*	0.35 ± 0.02	0.38 ± 0.01
Liver	2.95 ± 0.09	3.45 ± 0.16*	2.88 ± 0.05	3.10 ± 0.12
Spleen	0.17 ± 0.01	0.21 ± 0.00**	0.19 ± 0.01	0.22 ± 0.01**
Kidneys	0.71 ± 0.03	0.68 ± 0.03	0.73 ± 0.03	0.66 ± 0.02
Brain	0.57 ± 0.04	0.46 ± 0.04*	0.52 ± 0.14	0.53 ± 0.02

Data are expressed as mean ± SEM. SEM: Standard error of the mean; Con = control; Pb = lead acetate; Pb + Lip = lead acetate + *L. javanica* tea infusion; Lip = *L. javanica* tea infusion. *p < 0.05; **p < 0.01 compared to Con.

### Effects of *Lippia javanica* on brain antioxidant status

The non-enzymatic total antioxidant capacity was similar (*p* > 0.05) across all treatment groups. However, Pb exposure resulted in depletion of GSH (*p* < 0.05) compared to control and treatment with *L. javanica*, with and without Pb resulted in increased GSH (*p* < 0.05; *p* < 0.01) compared to the Pb group. For enzymatic antioxidant SOD, all groups exhibited increased SOD activity (*p* < 0.05) compared to control. The Pb exposed group had higher lipid peroxidation as shown by the higher MDA concentration compared to controls, while Pb + *L. javanica* and *L. javanica* treatment groups showed reduced MDA level (*p* < 0.05) compared to the control. Results are summarised in Table 2.

**Table 2:** Antioxidant status of brain tissue

Parameter	Treatment Groups			
	Con	Pb	Pb + Lip	Lip
TAC (mg/ml TE)	3.73 ± 0.0	3.57 ± 0.1	3.66 ± 0.0	3.62 ± 0.1
GSH (μMol /g tissue)	67.4 ± 1.8	57.1 ± 2.8*	63.2 ± 1.5^##^	75.8 ± 2.3^###^
SOD (U/mL)	16.2 ± 1.1	20.2 ± 1.4*	20.6 ± 1.3*	20.6 ± 1.5*
MDA (μMol/L)	3.81 ± 0.7	7.76 ± 0.5*	6.17 ± 0.2	4.67 ± 1.4

Data was expressed as mean ± SEM

*S.E.M* Standard error of the mean, *TAC* Total antioxidant capacity, *GSH* Reduced glutathione, *SOD* Superoxide dismutase activity, *MDA* Malondialdehyde, *Con* control, *Pb* lead acetate, *Pb + Lip* lead acetate + *L. javanica* tea infusion, *Lip L. javanica* tea infusion

*p < 0.05 compared to Con; ^##^*p* < 0.01, ^###^*p* < 0.001 compared to Pb.

### Effect of *Lippia javanica* on brain acetylcholinesterase activity

**Fig. 1:**
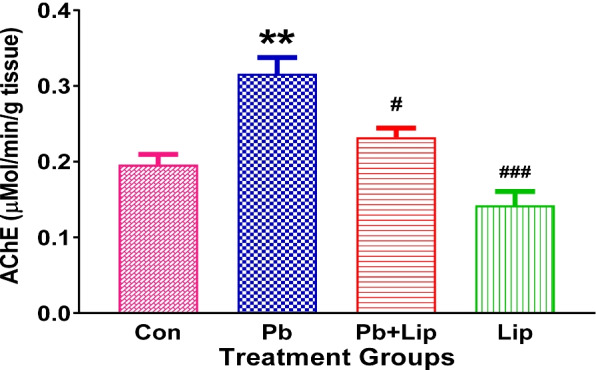
Effect of *L. javanica* treatment on acetylcholinesterase (AChE) activity in brain. Data was expressed as mean ± SEM. S.E.M: Standard error of the mean; Con = control; Pb = lead acetate; Pb + Lip = lead acetate + *L. javanica* tea infusion; Lip = *L. javanica* tea infusion; **p < 0.01 compared to Con; ^#^*p* < 0.05, ^###^*p* < 0.001 compared to Pb

Exposure to Pb resulted in increased AChE activity (*p* < 0.01) compared to control. This effect was mitigated by treatment with *L. javanica* which resulted in decreased AChE activity to levels similar with control group and lower than Pb group (*p* < 0.05). Treatment with *L. javanica* alone also reduced AChE activity compared to the Pb exposed group (*p* < 0.001) (Fig. 1).

### Effect of *Lippia javanica* on brain inflammation and apoptosis

**Fig. 2:**
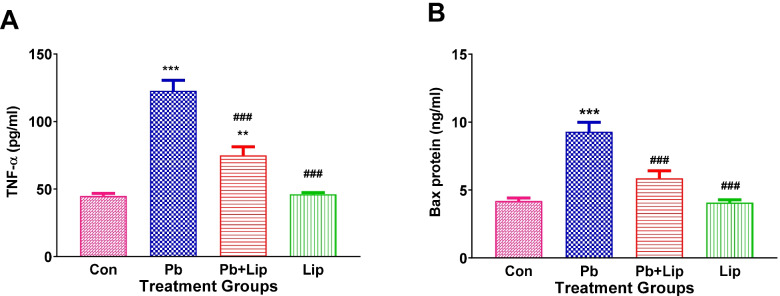
Effect of *L. javanica* treatment on pro-inflammatory cytokine TNF-α (**A**) and pro-apoptosis protein Bax (**B**) in brain tissue. Data was expressed as mean ± SEM. S.E.M: Standard error of the mean; Con = control; Pb = lead acetate; Pb + Lip = lead acetate + *L. javanica* tea infusion; Lip = *L. javanica* tea infusion; **p < 0.01; ***p < 0.001 compared to Con; ^###^*p* < 0.001 compared to Pb

Pro-inflammatory cytokine, TNF-α was highest in the Pb exposed group and was significantly higher (*p* < 0.001) than the control as well as the other treatment groups. Treatment with *L. javanica* partially reversed the Pb effects by reducing brain TNF-α concentrations compared to Pb group (*p* < 0.001) but remained higher (*p* < 0.01) compared to controls. Rats treated with *L. javanica* alone were similar to controls (Fig. 2A). Observations on the pro-apoptosis protein Bax were similar to those for TNF-α except that the Pb + *L. javanica* showed Bax levels lower than Pb group and similar to control. (Fig. 2B).

### Histological effects of *Lippia javanica* herbal tea

**Fig. 3:**
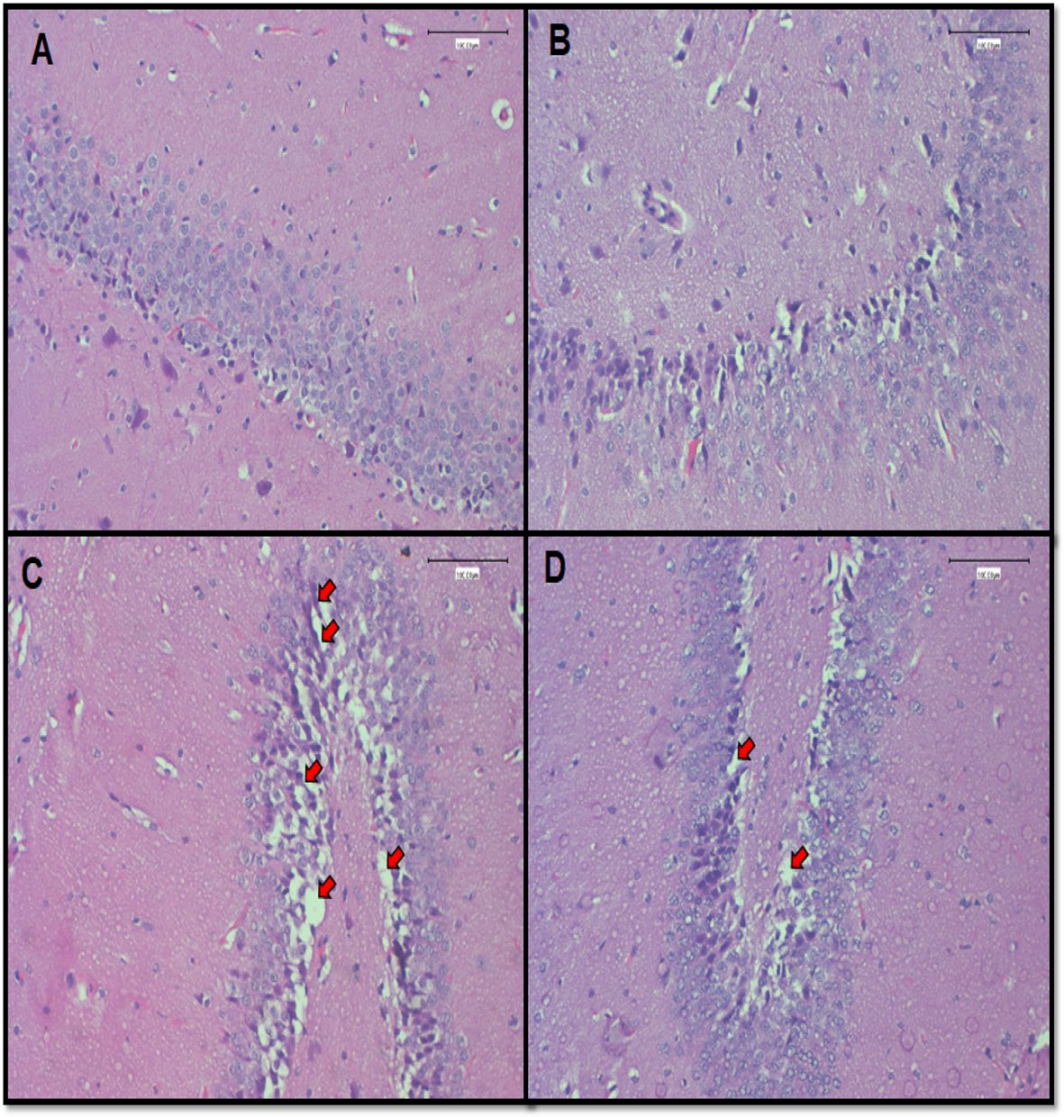
Photomicrographs of rat hippocampus sections H&E stained observed at × 20 magnification. Ruler bar = 100 μm. Arrows indicate vacuolation and oedema. **A** = Control; **B** = *L. javanica*; **C** = lead acetate; **D** = lead acetate + *L. javanica*

Histological study of the hippocampus from the control and *L. javanica* treated groups showed normal hippocampal structure with pyramidal cells arranged neatly and tightly with no noticeable vacuolation or oedema (Fig. 3A & B). However, the Pb exposed group showed severe vacuolation and oedema in the hippocampus, consistent with inflammation and cellular damage. Pyknotic nuclei are evident around areas of vacuolation (Fig. 3C). *L. javanica* treatment resulted in a clear improvement of Pb-induced hippocampal damage with less vacuolization and oedema presenting neuroprotective effects (Fig. 3D).

### Phytochemicals present in *Lippia javanica* revealed by LC-MS

*L. javanica* was shown to be rich in phenolics including syringaldehyde, protocatechuic acid, trans-cinnamic acid, syringic acid, caffeic acid, *p*-coumaric acid, ferulic acid, gallic acid and vanillic acid as shown in Table 1. The relative abundance in increasing order is as follows: vanillic acid (10.87%), > caffeic acid (14%), > *p*-coumaric acid (16.65), > protocatechuic acid (21%), > syringic acid (25.34%) with these five phenolics accounting for up to 88% of total phenolics identified in *L. javanica* (Table 3).

**Table 3:** Chemical composition of *Lippia javanica* detected by LC-MS

Compound	Concentration (μg/L)	Abundance (%)
Syringic acid	334.40 ± 24.2	25.34
Protocatechuic acid	277.23 ± 23.0	21.00
*p*-coumaric acid	219.67 ± 11.6	16.65
Caffeic acid	184.20 ± 8.5	13.96
Vanillic acid	143.38 ± 5.4	10.87
Trans-cinnamic acid	64.27 ± 5.3	4.87
Syringaldehyde	41.73 ± 2.9	3.16
Gallic acid	27.66 ± 1.3	2.10
Ferulic acid	27.23 ± 2.1	2.06

Values expressed as mean ± standard error of mean, *n* = 3

## Discussion

Pb-induced toxicity, particularly neurotoxicity is known to be mediated through impairment of cholinesterase activity, generation of ROS as well as pro-inflammatory cytokines which can also generate ROS. *L. javanica* has been reported to possess antioxidant activity to scavenge ROS [24]. However, it remains unknown whether the *L. javanica* can promote neuroprotective effects through its antioxidant activity against Pb-induced oxidative stress, inflammation, apoptosis and cholinesterase activity. This study was conducted to investigate the in vivo effects of *Lippia javanica (Burm.f.) Spreng* on Pb-induced brain oxidative stress and associated neurodegenerative effects.

Pb has the ability to cross the blood-brain barrier by competing with Ca^2+^ during neuronal firing and has been associated with neurodegenerative disorders [37]. It is well established that the neurodegenerative effects of Pb are linked to its role in causing oxidative stress [1, 6, 27]. As such, studies have looked at combating brain oxidative stress and neurodegeneration with antioxidants [38]. *Lippia javanica*, a herbal tea plant whose antioxidant activity has been studied almost exclusively in vitro [24, 39]*,* has not been investigated for antioxidant effects in vivo especially in the brain. To our knowledge, this is the first study to investigate effects of *L. javanica* on Pb-induced brain damage. To achieve this, we exposed rats to Pb to induce damage and observed the effect on the animal’s organs, paying attention to the brain which was our organ of interest. Brain weight was reduced as well as toxic effects on liver, heart and spleen were observed, and consistent with observations in previous studies attributed to Pb-induced oxidative stress, inflammation, fibrosis and cell death [40].

The brain is highly susceptible to oxidative damage and lipid peroxidation due to its high oxygen requirements and high lipid content. The levels of glutathione and α-tocopherol, both non-enzymatic antioxidants, are relatively low in the brain compared to the rest of the organs in the body [41]. Ingestion of plant-based antioxidants in the form of polyphenols may increase the brain’s antioxidant capacity. Faria and colleagues [42] demonstrated that flavonoids are able to cross the blood-brain barrier and showed intact anthocyanins within various brain regions. In our study, the TEAC assay was used to measure the overall non-enzymatic antioxidant status in the brain, which included hydrophilic and lipophilic antioxidants as well as –sulfhydryl-containing antioxidants. Pb exposure had no effect on overall antioxidant status in the brain, nor did *L. javanica*. However, glutathione was significantly increased in the *L. javanica*-treated Pb exposed animals compared to animals exposed to Pb only. These results suggest that though the total antioxidant level was same, *L. javanica* specifically increased glutathione level. Khalaf and colleagues [43] reported that Pb exposure at 100 mg/kg BW resulted in lowered antioxidant status in the brain while green tea consumption at 5 g/L ad libitum increased overall non-enzymatic antioxidant status in the brain, also using the TEAC assay.

SOD is an enzyme family that protects cells from the harmful effects of O^2−^ radical. In light of the brain’s low, non-enzymatic antioxidant status, enzymatic antioxidants such as SOD are extremely important in protecting neurons against free radicals and oxidative stress. The SOD status in the brain was increased with Pb exposure, *L. javanica* consumption as well as with *L. javanica*-treated Pb exposure. This increased SOD activity may have a protective role in the cells of the brain which, in the case of Pb exposure, may be a protective response while the increase of SOD status may be due to upregulation by *L. javanica* treatment. Again, in parallel with our results, Reckziegel and colleagues [17] reported increased SOD status with Pb exposure as well as with gallic acid treatment.

Lipid peroxidation (LPO) can be defined as a free radical oxidation of polyunsaturated fatty acids [44]. LPO, as determined by MDA concentration, was increased in the Pb exposed group indicating that Pb exposure led to LPO in the brain as has previously been demonstrated [45]. On the other hand, though Pb-exposed *L. javanica* treatment group had increased LPO, it showed a trend towards a decrease compared to Pb exposure alone group (*p* = 0.1) indicating that *L. javanica* treatment prevented LPO to an extent. This was confirmed as treatment with *L. javanica* had no effect on LPO as the MDA level was similar to controls. Similarly, Reckziegel and colleagues reported that MDA concentration was increased with Pb exposure, but treatment of Pb exposure with gallic acid, a known flavonoid, reduced Pb-induced LPO in the brain [17]. Also, curcumin has been shown to inhibit Pb-induced oxidative stress and chelating activity [46].

Pb exposure has been suggested to promote inflammation. Generally, inflammatory cytokine expression is very low or undetectable in the brain under physiological conditions but tends to increase in conditions such as infections, trauma, autoimmune diseases or exposure to toxic agents [47]. Pb toxicity has been shown to induce increase level of pro-inflammatory cytokines such as IL-6, TGF-β1 [11] and TNF-α [48]. Findings from this study showed that Pb induced inflammation as increased level of TNF-α was observed in Pb exposed animals but was reduced by *L. javanica* as the Pb exposed animals treated with *L. javanica* had a significantly lower TNF-α level. This finding confirms previous studies which have also shown some plants to prevent Pb-induced inflammation [49]. Pb toxicity is known to promote apoptosis as it has been shown to increase the expression and synthesis of some pro-apoptotic proteins such as bcl-2 [14] and Bax proteins [50]. Pb induce the synthesis of Bax protein as it significantly increased in Pb exposed animals but was reduced by *L. javanica* treatment suggesting that *L. javanica* can prevent Pb-induced apoptosis. Pb exposure has also been observed to affect neurotransmission. Pb has been shown to affect cholinergic systems in the hippocampus and septum impairing cholinergic nervous transmission [10]. Acetylcholinesterase regulates cholinergic neurotransmitters. Increased acetylcholinesterase activity impairs nervous transmission [11]. In this study, Pb exposed animals which were treated with *L. javanica* had a significantly reduced acetylcholinesterase level which was high in Pb exposed animals. Therefore, *L. javanica* treatment reduced cholinesterase activity which was induced by Pb. This finding is consistent with previous studies which have also shown *Thunbergia laurifolia* (Linn.) [51] and propolis [52] to attenuate Pb-induced cholinesterase activities in animal models.

The hippocampus is a bilateral brain structure located in the temporal lobe on which learning and memory are critically dependant [53]. Episodic memory impairment is a hallmark of neurodegenerative diseases, such as Alzheimer’s disease, which are neurobiologically linked to the hippocampus [54]. Liu and colleagues [48] reported that Pb exposure induced significant microgliosis and astrogliosis in hippocampus of young mice. In Pb treated rats, vacuolization and oedema was observed in the hippocampus area [43]. In our current study, Pb exposure caused severe hippocampal cellular damage which was similarly manifested as vacuolization and oedema. The hippocampal structure remained normal with *L. javanica* treatment, consistent with controls. The effects of Pb-induced hippocampal damage were decreased with *L. javanica*, similar to previously published studies using green tea [43]. Thus, *L. javanica* protects against Pb-induced neuronal damage.

To ascertain the phytochemicals in *L. javanica* that were responsible for the neuroprotective effects of Pb-induced brain damage, LC-MS analysis was done*. L. javanica* was shown to be rich in phenolic acids among which vanillic acid, caffeic acid, *p*-coumaric acid, protocatechuic acid and syringic acid were high in abundance accounting for about 88% of the total phenolics while gallic acid, syringaldehyde, trans-cinnanic acid and ferulic acid were in small amounts. This finding confirms previous reports which have shown *L. javanica* to be rich in polyphenols such as flavonols flavonoids, and proanthocyanidin [55, 56]. Most of these abundant phenolics compounds (syringic, *p*-coumaric, caffeic and vanillic acids) found in *L. javanica* have been reported in experimental models to possess anti-inflammatory and antioxidant activities [57–61] as well as anticholinesterase activity [62, 63] and in apoptosis in reducing the expression of Bcl [64]. This may suggest that the observed effects of *L. javanica* in this study may be accounted for by these phenolic compounds.

## Conclusions

This study demonstrated that *L. javanica* possess a neuroprotective effect as it was effective in reducing Pb-induced brain oxidative stress, inflammation, apoptosis, acetylcholinesterase activity and neuronal damage. Thus, *L. javanica* hearbal tea may be effective in preventing the onset of inflammation, apoptosis, oxidative stress-induced neurodegeneration, neuronal damage and its associated diseases.

## Data Availability

All data generated or analysed during this study are included in this published article.

## Abbreviations

ABTS: 2, 2′-azino-bis-(3-ethylbenzthiazoline-6-sulphonic acid
AChE: Acetylcholinesterase
Bax: Bcl-2 Associated X Protein
CAT: Catalase
CDC: Centers for Disease Control
CNS: Central nervous system
LC-MS: Liquid chromatography-Mass spectrometer
GPX: Glutathione peroxidase
GR: Glutathione reductase
GSH: Glutathione
GSSG: Oxidised state
H_2_O_2_: Hydrogen peroxide
HO_2_: Hydroperoxides
IL: Interleukin
LPO: Lipid peroxidation
MDA: Malondialdehyde
Pb: Lead
ROS: Reactive oxygen species
SOD: Superoxide dismutase
TAC: Total Antioxidant capacity
TBARS: Thiobarbituric acid reactive substances
TE: Trolox equivalent
TEAC: Trolox equivalent antioxidant capacity
TGF-β1: Transforming growth factor β1
TNB: 5- thionitrobenzoic acid
TNF-α: Tumour necrotic factor-α
